# The Development and Pilot Testing of a Fidelity Checklist for a Family-Centered Telehealth Intervention for Parents of Children with Motor Delay

**DOI:** 10.5195/ijt.2024.6603

**Published:** 2024-06-28

**Authors:** Karen Hurtubise, Michelle Phoenix, Chantal Camden, Raphaëlle Gauthier, Paul Stratford, Rosalie Dostie, Audrée Jeanne Beaudoin, Désirée Maltais, Jade Berbari, Isabelle Gaboury

**Affiliations:** 1 School of Rehabilitation Sciences, Faculty of Health Sciences, McMaster University, Hamilton, Ontario, Canada; 2 Faculté de Médicine et Sciences de la Santé, Université de Sherbrooke, Sherbrooke, Québec, Canada; 3 CanChild Centre for Childhood Disability Research, McMaster University, Hamilton, Ontario, Canada; 4 Centre de recherche du Centre Hospitalier Universitaire de Sherbrooke, Sherbrooke, Québec, Canada; 5 École de sciences de réadaptation, Faculté de Médecine, Université Laval, Québec, Québec, Canada; 6 Centre interdisciplinaire de recherche en réadaptation et intégration sociale, Québec, Québec, Canada

**Keywords:** Fidelity measure development, Implementation experience, Pediatric rehabilitation, Telehealth

## Abstract

This multi-methods study describes the development of a pediatric rehabilitation telehealth intervention fidelity checklist, estimates its inter-rater reliability, and documents raters' implementation experience. A literature scan and expert consultation identified eighteen key behaviors and categorized them into three subdomains, measured using a 5-point measurement system. To estimate the checklist's inter-rater reliability, three raters scored 33 video recordings. A Shrout and Fleiss Class 1,1 intraclass correlation (ICC)) and 95% confidence intervals (CI) calculated ICCs = 0.5 (CI: 0, 0.9) for both the Therapist and the Parent-Therapists subdomains, and the Parent subdomain an ICC = 0.3 (CI: 0, 0.8). In the implementation surveys, raters reported high levels of satisfaction (100%), ease of use (84% to 88%), and confidence in their video ratings (87% to 100%). Changes in procedures and scoring were recommended. Capturing raters' implementation experiences is crucial in the early evaluation of the fidelity checklists for telehealth.

Intervention fidelity is a multidimensional construct which refers to the faithfulness and accuracy to which an intervention is implemented. The Implementation Fidelity Framework (Carroll et al., 2007) outlines potential therapist behaviours, client features independent of the therapist, and client attributes that are dependent on the therapist (therapist-client interaction) which can impact the intervention fidelity (DiRezze et al., 2012). Based on this framework, therapist adherence in delivering the intervention mediates intervention fidelity, while the quality of the therapy delivery and participant responsiveness are moderators of the clinician and the recipient's behaviors in the intervention (Carroll et al., 2007). Adherence measures the accuracy of the delivery of the key components of the intervention as it was designed (Gresham et al., 1993; Moncher & Prinz, 1991). The quality of the intervention delivery is related to how therapists implemented the intervention using the overall process and strategies designed by its developers (Gresham et al., 1993; Moncher & Prinz, 1991). Participant responsiveness involves the extent to which participants respond to or engage in the intervention (Gresham et al., 1993; Moncher & Prinz, 1991). Adequate assessment and reporting of the intervention fidelity dimensions ensure a truthful representation, implementation, and evaluation of an intervention, and facilitate its replicability in future studies and alternate settings (Carroll et al., 2007). Although fidelity is considered an important aspect to measure, studies measuring intervention fidelity are rare in the field of pediatric rehabilitation (An et al., 2021) and even rarer in interventions delivered via telehealth. Fidelity evaluations often focus only on adherence, neglecting the moderator dimensions (An et al., 2020). Moreover, fidelity can be complex to study in pediatric rehabilitation owing to the many factors that can influence therapists' performance in delivering an intervention (DiRezze et al., 2012).

When a new intervention is introduced, a fidelity process needs to be developed that reflects the key ingredients of the novel intervention, without which intervention success would be compromised, and inaccurate interpretation of treatment effects could ensue (An et al., 2020; Gearing et al., 2011). In the case of this study, the new intervention was the Web-based Early intervention for Children with motor difficulties using multimodAl REhabilitation (*WECARE*) intervention. The *WECARE* intervention was developed for a family-centered pragmatic randomized control trial (NCT04254302) comparing its effectiveness to usual care on the functional abilities of children (aged 3-8 years) with motor difficulties and their parents' knowledge, skills and health-related quality of life (Camden et al., 2021). The intervention was created based on the findings of previous pilot and feasibility studies, a systematic review of pediatric rehabilitation telehealth interventions, and consultations with parents, clinicians, and other interested parties (e.g., managers, technicians) who may be involved in such an intervention (Camden et al., 2019; Camden et al., 2020; Hurtubise et al., 2022; Pratte, 2018). This intervention was delivered entirely via a multimodal web-based platform. Parents of children (aged 3-8 years) with or at risk of a motor delay (as determined by the DCD-Q or Little DCD-Q) were assigned a primary therapist, either a pediatric occupational therapist or a physiotherapist. *WECARE* was grounded in a transdisciplinary collaborative family-centered coaching approach, delivered by the therapists via telehealth, which aimed to support the participating parents in helping their child to reach meaningful motor goals, as established, and prioritized by the parent. Parents and therapists met via the platform's videoconferencing feature for 30-minute appointments, every two weeks for the first three months, as a means of establishing a therapeutic alliance between the therapist and the parent, and for parents to become familiar and engaged with the coaching process. Following the initial 3 months, the appointment times and frequency were adapted based on parents' identified needs and at parents' request until they completed their participation in the study. As flexibility is inherent to coaching approaches, no strict schedule of coaching sessions was instituted, allowing time between sessions for parents to work on their goals via practice with self-selected strategies and activities.

As the provision of coaching interventions in pediatric rehabilitation using telehealth is a novel approach, assessing fidelity for this type of intervention is in its infancy and often an overlooked component of the intervention evaluation (Ward et al., 2020). Moreover, existing fidelity evaluation instruments are not optimal. For instance, the Paediatric Rehabilitation Observational measurement of Fidelity (PROF), although a reliable and valid fidelity instrument, is a generic measure designed to evaluate interventions specifically targeting children with cerebral palsy (DiRezze et al., 2013). Owing to its generic nature, the PROF is not well-suited to study the active intervention ingredients specific to the novel *WECARE* intervention (DiRezze et al., 2012). Furthermore, the PROF was designed to evaluate child- and context-focused interventions, as compared to parent-targeted interventions, such as coaching, as is the case with the *WECARE* intervention (DiRezze et al., 2013). In contrast, the Checklist for Implementing Parent-Delivered Therapy Intervention (Lord et al., 2018), a quick and accessible instrument when initiating a parent-delivered intervention, can be adapted and tailored to specific interventions, but it is still a prototype, has not yet been standardized, and, as a result, requires further study before implementation. Finally, neither the PROF nor the Checklist for Implementing Parent-Delivered Therapy Intervention have been tested in the context of telehealth service delivery.

To our knowledge at the time of the launch of the *WECARE* intervention, only one coaching-specific intervention fidelity measure, the Occupational Performance Coaching Fidelity Measure (OPC-FM) (Graham et al., 2020), had been applied in the telehealth context. As an intervention-specific fidelity measure, this instrument examines ingredients key to the intervention for which it was designed (i.e., Occupational Performance Coaching) (Dunn et al., 2018). Although this intervention shares some similar features, other key ingredients are unique to the *WECARE* intervention and are not represented in this instrument.

Like the intervention they seek to evaluate, developing succinct fidelity instruments can be complex, time-consuming and onerous (Ginsburg et al., 2021). The use of observation and checklist tools, which do not rely on study participants' responses can improve the quality of the data used for intervention fidelity assessments (Ginsburg et al., 2021). Consequently, this article aims to describe the process conducted to develop the intervention-specific *WECARE Fidelity Checklist* and the results of the assessment of its initial implementation.

The checklist was one of several methods used for assessing the fidelity of implementing the videoconferencing coaching component of the *WECARE* intervention. More specifically, this checklist aimed to determine if the therapists involved in implementing the *WECARE* coaching intervention were doing so as intended (i.e., adherence), using the overall strategies outlined by the study designers (i.e., quality of the intervention), as well the extent to which participants responded to and engaged in the intervention activities (i.e., participant responsiveness) (Kutash et al., 2012). As the administration of a new instrument can be challenging, its success can depend on multiple factors, such as processes (e.g., training in the instrument use, ease of the rating procedure), measurement characteristics (e.g., clarity of the behavior definition, rating scales), and the people administering it (i.e., the raters). Understanding the factors impacting an instrument's administration and its implementation, as well as its utility are important components for its refinement and its potential future adoption (Duncan & Murray, 2012). As a result, in addition to detailing the checklist development process, this article will also estimate the inter-rater reliability and describe the external raters' experiences in using the new fidelity measure.

## Methods

### Study Design

**Figure 1:**
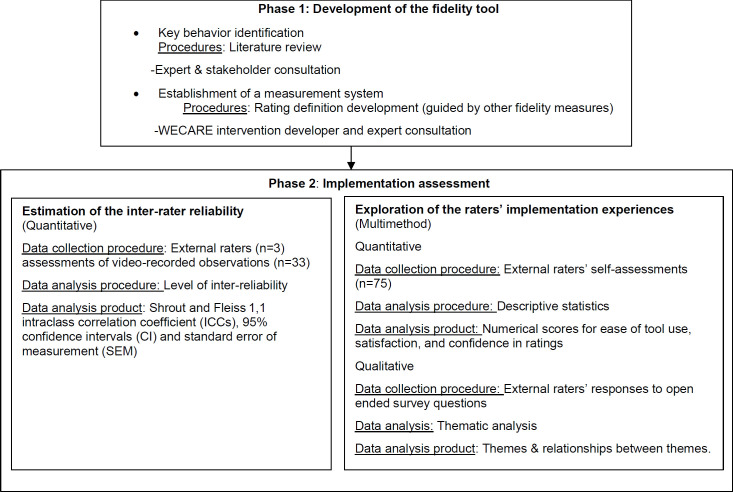
Summary of the Study Design

This study was nested within the context of the *WECARE* family-centered pragmatic randomized control trial (Camden et al., 2021). It used a multi-methods approach. Figure 1 illustrates the study design, highlighting the sequence of research phases and activities, along with the data collection and analysis methods employed during each phase. Ethical approval was received from the institutional ethics committee (*Centre intégré de santé et des services sociaux de l'Estrie—Centre hospitalier universitaire de Sherbrooke*; identifier: 2020-3429). Informed consent was obtained from raters.

### Study Context

The WECARE study was launched in the province of Quebec, Canada, four days before the provincial government declared a public health state of emergency due to the COVID-19 pandemic. Provincial public health lockdown measures were enforced from March 2020–June 2021 (Gouvernement du Québec, 2021). As part of these lockdown measures, schools, outpatient pediatric rehabilitation services, and non-essential workplaces and businesses were intermittently closed (Berasategi Sancho et al., 2022). Parents transitioned to work-from-home situations or were redeployed to essential services. Children attended virtual classrooms and/or learning environments. Although the WECARE study was planned and funded before COVID-19, these lockdown conditions were the contextual reality in which the *WECARE* intervention was delivered for the duration of the study.

### Phase 1- Development of the Fidelity Tool

The intervention-specific fidelity checklist development was influenced by the process described by An and colleagues (An et al., 2020). The process began with (1.1) the identification of the key components of the *WECARE* intervention, followed by (1.2) the establishment of the measurement coding system (i.e., specific assessment criteria as it related to key components and how to determine if the component was implemented with the appropriate level of fidelity).

#### 1.1 *Identification of the key intervention components:*

The key components defined the focus of the intervention, as well as the portion of the intervention that, if removed, would change it completely (An et al., 2021). Because rehabilitation is a dynamic and interactive process, many interventions will have common components (An et al., 2021). Based on the theory of change supporting the *WECARE* intervention (Camden et al., 2021; Hurtubise et al., 2022), this exploratory step was comprised of (a) a scan of the literature, and (b) validation of the components by experts' opinion. The rapid scan examined empirical evidence related to coaching interventions used in pediatric rehabilitation (Akhbari Ziegler & Hadders-Algra, 2020; Baldwin et al., 2013; Dunn et al., 2018; Little, Pope, et al., 2018; Ogourtsova, O'Donnell, et al., 2019; Ogourtsova, O'Donnell, et al., 2019; Schwellnus et al., 2015), pediatric telehealth (Camden et al., 2020; Camden & Silva, 2021; Cason et al., 2012; Little et al., 2018; McCarthy & Guerin, 2021; Olsen et al., 2012), family-centered care practices (An & Palisano, 2014; Bamm & Rosenbaum, 2008; King, 2014; King et al., 2017; Law et al., 2003), parent engagement (King et al., 2019; King et al., 2014), and measures related to parent engagement, and generic and coaching specific intervention fidelity in pediatric rehabilitation (Dunn et al., 2018; Graham et al., 2020; King et al., 2019; DiRezze et al., 2013).

Based on literature findings, two members of the *WECARE* research team (KH, a postdoctoral fellow and clinician with experience delivering pediatric rehabilitation services via telehealth, and CC, the principal investigator) generated a list of key observable behaviors. These behaviors were then discussed with two experts, an experienced pediatric physiotherapist and expert in delivering coaching interventions and establishing intervention fidelity, and an experienced pediatric occupational therapist, with expertise in providing and studying parent coaching. These discussions aimed to ensure that components corresponded to coaching practices in pediatric rehabilitation, that all key components were included, and that none were inadvertently omitted.

#### 1.2. *Establishment of a measurement coding system:*

Determining how to quantify the key components is the next step in the fidelity measure development (An et al., 2021). A 5-point rating scale was chosen due to its simplicity, efficiency, and the multiple categories of behaviors (i.e., therapists, parent, and parent-therapist interactions) explored, (Chyung et al., 2017). To establish if the intervention was delivered with an acceptable level of fidelity (i.e., adherence, quality, and participant responsiveness), rating criteria were based on whether the behavior was present or absent (Waltz et al., 1993), and whether it was used appropriately (or not) for a given situation (Dunn et al., 2018; Graham et al., 2020) (i.e., 0=absent; 5=always present).

Once the key behaviours were validated, an initial draft of definitions for each scale level was created by KH, and refined by CC, following which, the criteria and their definitions were applied to clinical scenarios, and further discussed with the same two experts involved in the key behaviour validation process. Details about the checklist administration method and process, frequency and timing of the assessments, and checklist measurement scoring system ensued.

As a result of these initial development steps, the *WECARE Fidelity Checklist* contained three ratable subscales. The checklist's cover page includes descriptors and definitions of each of the rating indicators and definitions. Dedicated space was provided for raters to record specific observations (e.g., technology or contextual disruptions) impacting rating, to provide justification for the assigned scores if required, and to document the presence of behaviors to be avoided or excluded from the intervention. This version of the measure was shared with the therapists implementing the *WECARE* intervention during their training.

### Phase 2-Implementation Assessment

As presented in Figure 1, this phase consisted of two components: (2.1) an estimation of the inter-rater reliability of the developed tool, and (2.2) the exploration of the external rater's experiences using the measure. The data collection and analysis procedures for each of these steps are explained in the following paragraphs.

#### 2.1. *Inter-rater reliability estimation:*

In piloting the checklist's measurement properties, we chose to estimate its inter-rater reliability, a psychometric property rarely examined in fidelity measures (Ginsburg et al., 2021). More specifically, the inter-rater reliability of the *WECARE Fidelity Checklist*'s three subscales were estimated within the context of the *WECARE* study. Feasibility studies examining reliability recommend a minimum of 24 files be utilized during the pilot testing of the instrument development phase (Bellg et al., 2004). We planned to have 33 video tapes reviewed by the same two raters. We estimated 33 videos based on the following assumptions: 95% confidence interval, two ratings per video; an expected reliability of 0.85; a confidence interval width of 0.20 (i.e., ±0.10) (Bonett, 2002).

##### 2.1.1. *Raters and training procedure:*

Two experienced pediatric therapists (i.e., an occupational therapist and a physiotherapist), each with over 10 years of clinical expertise and training in delivering parent coaching interventions, acted as the external raters (i.e., Rater 1 and Rater 2). Both raters were also involved as experts in validating and finalizing the key behaviors and the rating scale level criteria and definitions. As part of the rater's training process, Rater 1 and CC independently coded the same video-recorded *WECARE* session, following which scoring results were compared and discussed. Rater 2 was provided with a written guide of the rating procedure, the rating criteria, and definitions, following which she also independently viewed and scored the same *WECARE* video-recorded session as Rater 1 and CC; results were then compared. Due to unforeseen circumstances, Rater 1 was unable to complete all assessments. Accordingly, a third rater was recruited to complete the assessment of the remaining videos. Rater 3, a pediatric physiotherapist with 5 years of clinical experience and limited experience in providing parent coaching intervention, was trained in applying the *WECARE Fidelity Checklist* using a process similar to that of Rater 2.

##### 2.1.2. *Data collection procedures:*

Each therapist providing the *WECARE* intervention was asked to record all parent-therapist videoconference sessions during the first week of each month of the study. One session was then randomly selected by a member of the research team from the bank of video recordings for evaluation by the external raters. Each rater independently reviewed the entire recorded session (which ranged from 24-63 minutes) and rated the items of the *WECARE Fidelity Checklist* subscales on the 5-point Likert scale. Reviewing the entire video ensures that crucial information is not missed as can be the case when video segments of a session are used (Gardner, 2000). In total, 33 videos were assessed. Of the 33 videos, Rater 2 evaluated all 33 videos, Rater 1 assessed 19 videos, and Rater 3 rated 20 recordings (i.e., the 14 remaining videos + 6 which overlapped with Rater 1 and Rater 2).

##### 2.1.3. *Data Analysis:*

The subscale scores were treated as interval data, given they were obtained by summing item scores. We chose to use intraclass correlation coefficients (ICCs) as our reliability coefficient, as they are widely used when assessing inter-rater reliability (Koo & Li, 2016). Given the rater pairings were not common to all videos as initially planned, we chose to calculate a Shrout and Fleiss Class 1,1 intraclass correlation (ICC) (Shrout & Fleiss, 1979). A Class 1,1 ICC is appropriate when either there is no common structure—as per the raters in this study—linking the measurements, or the number of ratings differs among the objectives of the measurement (Shrout & Fleiss, 1979). For measures in the early stages of development, as is the case with *WECARE Fidelity Checklist*, claims of acceptability should be accompanied by a rationale specific to the tool being assessed (Portney, 2020)*,* We also calculated the 95% confidence interval (CI) and standard error of measure (SEM), which quantifies measurement error in the same units as the original measurement. Finally, the instrument is comprised of behavioral items that define the features of the various components of the *WECARE* intervention. As explained by Streiner (2003) in such a case, the coefficient alpha is not relevant; all relevant items must be included and there is no expectation that the items would correlate with each other.

#### 2.2. *Exploring raters' implementation experience:*

Qualitative and quantitative data were collected to gain a more robust and clear understanding of the external raters' experience in implementing the *WECARE Fidelity Checklist* tool, and the factors impacting its implementation.

##### 2.2.1. *Data collection procedures:*

A rater experience survey was created for the study, which evaluated the following three aspects of the raters' experience in implementing the tool on a 5-point Likert scale: satisfaction (0=not at all satisfied to 4=very satisfied) and global experience (0=very difficult and 4=very easy) with the tool, as well as the confidence level with their ratings (0=not confident at all to 4=very confident). After scoring each video, the external raters were asked to complete the survey. Open space was available at the end of the survey for raters to further describe their experience with the tool, along with their recommendations for improvements. Seventy-two surveys were completed.

##### 2.2.2. *Data analysis procedure:*

Descriptive statistics were used to analyze the survey response (i.e., Likert scale) data. A pragmatic iterative inductive thematic analysis approach was used to analyze the comments included in the open space of the survey (Saldaña & Omasta, 2016). Two members of the research team, KH and a physiotherapy student (RG), both trained and experienced in thematic analysis, performed the initial thematic coding. First, both coders independently reviewed and descriptively coded raters' comments related to the raters' experience and their recommendations for improvement (Saldaña & Omasta, 2016). They then met to discuss initial codes and reconcile differences; codes were categorized and subsequently themed and defined. To maintain rigor, an established method (Saldaña & Omasta, 2016) was utilized, with discussions and communication occurring regularly between the two coders. Member checking was also employed (Tracy & Hinrichs, 2017). As such, the identified themes and their definitions were sent to all raters for review and comment to ensure that the coders' interpretation of their experience was appropriate.

## Results

### Phase 1. Fidelity Tool Development

#### Key Intervention Component Identification

**Figure 2:**
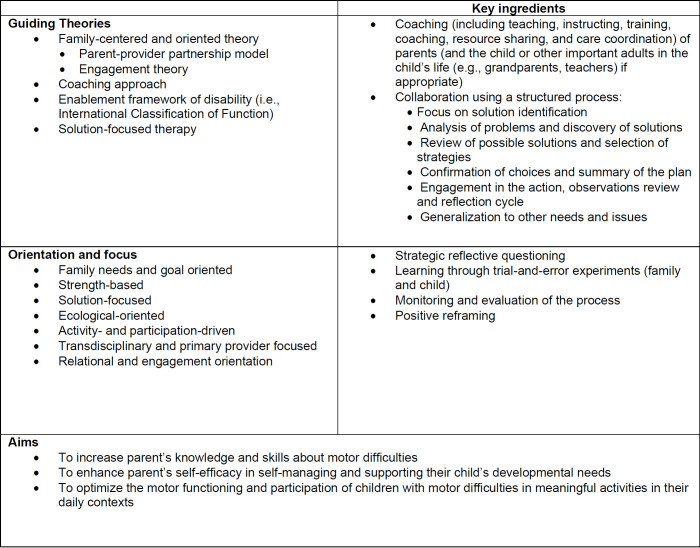
Characteristics of WECARE Family-centered Telehealth Coaching

The process of identifying key components of the *WECARE* intervention was informed by the theory of change supporting the *WECARE* intervention (Camden et al., 2021; Hurtubise et al., 2022), the Paediatric Rehabilitation Observational Measure of Fidelity (PROF) parent and therapist-parent behaviors (DiRezze et al., 2013), the Pediatric Rehabilitation Intervention Measure of Engagement (PRIME-O) observational measure of therapists, parent, and therapist-parent interaction behaviors (King et al., 2019), and the Occupational-Performance Fidelity Measure (OPC-FM) (Graham et al., 2020). These key intervention components created the foundation for the *WECARE Fidelity Checklist* (see Figure 2 for these components). Moreover, the key component list comprised observable behaviors associated with the therapist, the parent, and the parent-therapist interaction.

Following discussions with experts, 18 behaviors and their operational definitions formed the basis of the fidelity measure and included twelve therapist behaviors, four parent behaviors, and two parent-therapist interaction behaviors.

##### 1.2. *Establishment of a measurement coding system:*

The selection of scaling for each component was based on examining fidelity as a degree of frequency and quality, rather than a dichotomous concept of whether a behavior was present or absent (Breitenstein et al., 2010; Carroll et al., 2007). As summarized in Table 3, we chose to combine these components into descriptions associated with a 5-point rating scale. DiRezze and colleagues (2013) found that quality ratings for a subscale may not be importantly different from frequency ratings.

**Table 1:** Fidelity Tool Scoring System

Score	External Rater description qualifiers
1	The behavior was not observed when it should have been.
2	The behavior is observed at a limited frequency and/or is only partially observed.
3	The behavior is observed at times, but several opportunities to use this behavior are missed.
4	The behavior is observed on a regular basis, but not all opportunities are seized.
5	The behavior is always observed when appropriate (i.e., is applied as intended in the development of the intervention and observed at the appropriate time).

Moreover, as highlighted in the description, the criterion is not only based on whether the behavior occurs or not (i.e., “occurrence and non-occurrence approach” (Waltz et al., 1993)) but also if it occurred at the appropriate frequencies and at the appropriate time, an indicator of occurrence quality. The summed score of each intervention-specific subscale within the fidelity measure indicated the degree of fidelity of each subscale. Based on this scoring system, for the therapist behavior subscale (i.e., 12 items) a minimum score of 12 and a maximum score of 60 was possible; for the parent behavior subscale (i.e., four items), a minimum of 4 and a maximum of 20 was attainable; and for the parent-therapist interaction subscale (i.e., two items) a minimum of 2 and a maximum of 10 was achievable. Higher scores were representative of better fidelity.

### Phase 2. Implementation Assessment

Seventy-two *WECARE Fidelity Checklists* assessments were returned complete (i.e., no missing data). These assessments were conducted on 33 videos; 27 videos were rated by two raters and six were assessed by all three raters. All data was included in the analysis for the inter-rater reliability estimation.

#### 2.1. Inter-rater Reliability Pilot Testing

The inter-rater reliability estimation results are shown in Table 2.

**Table 2:** Inter-rater Reliability Analysis Summary

	Therapist Domain	Parent Domain	Parent Therapist
Scale: Min., Max.	10, 60	4, 20	2, 10
ICC (95% CI)	0.5 (0, 0.8)	0.3 (0, 0.8)	0.5 (0, 0.9)
SEM (95% CI)	6.0 (4.8, 7.9)	1.9 (1.5, 2.5)	1.1 (0.9, 1.5)

The assigned scores spanned the full range of the measurement system for each subscale. The ICCs for two of the subscales (i.e., Therapist and Parent-Therapist Domains) were 0.5 and when interpreted alongside the 95% CI revealed considerable uncertainty. Finally, it should be noted that the SEM is approximately 10% of the maximum possible value for each of the subscales. To further assess these discrepancies, secondary analyses examined the videos (n=6) rated by all three raters.

Upon closer examination of the data, particularly high variations among rater scores were noted in one video, with Rater 3 assigning lower scores as compared to Rater 1 and 2. When this video was removed, the interrater reliability improved in all subscales, yet considerable uncertainty remained (see Table 3).

**Table 3:** Inter-rater Reliability Analysis (Subsample)

6 video recordings	Therapist Domain	Parent Domain	Parent Therapist
Scale: Min., Max.	10–60	4–20	2–10
ICC (95% CI)	0.5 (0, 0.9)	0.3 (0, 0.8)	0.5 (0, 0.9)
SEM (95% CI)	7.1 (4.9, 12.4)	1.4 (1.0, 2.6)	1.0 (0.7, 1.0)

5 video recordings	Therapist Domain	Parent Domain	Parent Therapist
Scale: Min., Max.	10–60	4–20	2–10
ICC (95% CI)	0.8 (0.4, 1.0)	0.5 (0, 0.9)	0.9 (0.5, 1.0)
SEM (95% CI)	3.8 (2.6, 7.4)	1.2 (0.8, 2.2)	0.5 (0.3, 1)

#### 2.2. The Implementation Experience of the Raters

##### 2.2.1.1. *External Rater Experience Survey Responses:*

There were high levels of positive experience using the survey reported by the three raters, as presented in Table 4. Raters were “very satisfied” with their global experience in using the tools in 100% of videos, they found the tools “very easy” to use on 84% to 88% of videos and were “very confident” on their ratings in 87% to 100% of videos.

**Table 4:** Survey Results

	Rater 1 (n=19)	Rater 2 (n=33)	Rater 3 (n=20)
Global experience = “satisfied + very satisfied”	19	33	20
Ease of use = “easy + very easy”	16	29	19
Confidence in scores = “very confident”	19	29	20

It should be noted that lower ratings were recorded by each rater related to their global experience using the tool; Rater 1 and Rater 2 recorded three lower ratings (3/19 and 3/33 respectively), while Rater 3 recorded only one (1/20). For Raters 1 and 2, these ratings occurred predominantly early in the study, and on one occasion, were associated with raters assessing the same video. In the case of Rater 3, the lower rating occurred midway through the study and was associated with a specific video also rated lower by Rater 1. External rater written comments provided some insights into these lower ratings.

##### 2.2.1.2. *External Rater comments:*

Two main factors were identified as impacting the rater's experience with the measurement tool and its associated process. These included: (a) learning to use the tool; (b) dealing with procedural issues. Important recommendations to improve the instrument and the procedures surrounding its use were identified.

Firstly, raters highlighted an initial learning process associated with the use of the *WECARE Fidelity Checklist* tool as it related to the interpretation of the items and the associated scoring system. Some confusion related to specific checklist items did emerge, which raised concerns about the interpretation of the items and the resulting ratings, as expressed by this rater:

“Certain behaviors are present in more than one item; is this purposeful? This could lead to “penalizing” [the therapists] twice” [Rater 1].

Raters also highlighted two specific items that conflicted with best practice (i.e., item 4, item 10). More specifically, raters questioned the wording of item 4 which reads as follows: *The therapist agrees with the parent-identified centered objectives that will guide the session***.** Raters highlighted that the current phrasing suggests that session objectives appeared to be the responsibility of therapists, instead of a collaborative effort between the therapists and the parent as suggested by current evidence. Raters also queried item 10 for similar reasons. That statement reads as follows: *The therapist gives advice, information, or references, only when asked by the parent to do so.* Again, raters acknowledged that the statement failed to represent the type of partnership suggested in the literature, in which the parent and the therapist collaborate and share decision-making.

As expected, with the repeated use of the checklist tool, raters' comfort increased, and a better understanding of the items and the rating process was acquired. In their comments, raters noted that time, practice, and repetition were required to become familiar with the tool, the rating system, and associated behaviors. Comfort was established once 3-5 video recordings were rated. Moreover, raters preferred to rate multiple video recordings in one session, which helped to gain familiarity with the behavior rating definitions and an understanding of the various behaviors.

However, raters also raised concerns about developing a bias if they rated multiple recordings in succession. This concern was raised about the viewing of “extreme sessions” (i.e., a session scoring very high or a session scoring very low) and its influence on the scoring of subsequent sessions. This rater provided the following reflection:

“I was very impressed by this session. I was possibly biased positively. I don't think I have ever rated a session so high [for its fidelity]” [Rater 3].

With time, raters described the tool and scoring system as easy to use when the session was appropriately structured and followed intervention guidelines. However, procedural issues could complicate and impact the rating experience, despite the ease of use of the tool. These issues included technical issues and the routine absence of key checklist behaviors, and if the rating process was interrupted for any length of time.

Technical issues associated with the quality of the recording were by far the most frequently acknowledged challenge and had the greatest impact on the rating experience. Of the 33 videos, raters reported technical issues in 11 (i.e., 33%) recordings that impacted the data that was available to the raters for fidelity scoring. Examples of these issues included poor sound quality, unpleasant background noise, video streaming interruptions (e.g., frozen screen), access to audio data only, the inability to see both the therapists and the parents on the screen at the same time. This rater described it like this:

“Very long session (63 minutes) without video (audio recording only). I did my best to rate, but much more difficult due to the absence of the non-verbal language and the large amount of information discussed” [Rater 2].

Raters also underscored challenges when certain key behaviors were not present or not observed regularly. Moreover, this was further complicated if the absence of the behavior was clinically relevant and was appropriately managed from a family-centered perspective. This quote provides an example:

“It is difficult to give a high-fidelity score when the clinician does not ask the parent for permission to provide a suggestion or advice. However, this seems to be done respectfully with the mother and not in an imposing way” [Rater 3].

As a result of a similar situation this rater provided the following insights:

“Should we adjust the checklist for follow-up sessions (without a clear objective) or do we accept that the rating will be low because some checklist behaviors are not present given that they are related to objectives not defined at the beginning of the meeting?” [Rater 2].

These comments highlighted a need to carefully consider the importance and subsequent weighting of objective identification for each session in the fidelity measure or if different criteria should be considered for follow-up sessions as compared to the initial session.

Additionally, raters also suggested other ratings be included in the scoring system, such as “not applicable and/or “unable to rate”, as explained by this rater's quote:

“Not possible to rate subscale 2 because of the image quality. So, I gave a 0. There needs to be another choice for these situations where the evaluator cannot do the evaluation (missing data) in my opinion” [Rater 1].

To compensate for missing ratings or when raters felt unsure of their ratings, detailed observations were included in the designated open space in the measure to provide justification. These descriptions provide the information they used to recognize key behaviors and to identify when therapists missed opportunities to integrate key behaviors. Furthermore, these details also documented raters' interpretations of verbal and non-verbal behaviors, and what data was used to judge the quality of the parent-therapist relationship (e.g., exhibiting mutual respect), of their collaboration (e.g., goal setting, identifying strategies and action plan), their communication (e.g., active listening, mutual sharing of ideas), and the balance of power between the therapist and the parent.

## Discussion

This study aimed to describe: (1) the process conducted to develop a novel intervention-specific fidelity tool (i.e., the *WECARE Fidelity Checklist*) to evaluate the fidelity of the *WECARE* family-centered telehealth intervention provided to parents of children with or suspected of motor delays (Phase 1), and (2) the implementation experience (Phase 2). By using the *WECARE Fidelity Checklist*, we aimed to identify if key elements of the videoconferencing component of the *WECARE* intervention were implemented as planned.

### Phase I- Development of the Fidelity Tool

The development of the multidimensional measure began with identifying the key ingredients of the interventions which aligned with family-centered care and coaching principles, and other evidence associated with pediatric rehabilitation service delivery. The identified items recognize the importance of the dynamic nature of the parent's and the therapist's behaviors, and their influence on the parent-therapist interaction within the intervention. Incorporating and evaluating these behaviors acknowledges the critical roles of both the parent and the therapist in an intervention session (DiRezze et al., 2013), as well as the vital contribution the therapist-parent interaction plays on a child's rehabilitation and developmental outcomes (An et al., 2016; King, 2017).

In addition to targeting the parent, the therapist, and the interactions between them, the specific *WECARE Fidelity Checklist* items were based on the observation of therapists' coaching behaviors, parent engagement behaviors, and parent-therapist collaborative interactive behaviors. The use of direct fidelity assessment methods such as video-observations and an associated checklist improves the accuracy of the fidelity assessment, and minimizes altered perception of previous performance, recall bias, or distortions of self-representation (Bayona et al., 2006). The therapist and parent behaviors align with the therapist and parent/client change mechanism recently identified in a comparison of three well-known evidence-based coaching approaches used in pediatric rehabilitation (i.e., Solution-Focused Coaching, Occupational Performance Coaching, Coping with and Caring for Infants with Special Needs) (King et al., 2023). More specifically, the authors of these coaching approaches acknowledged that therapists' change mechanisms included non-directive, collaborative, and reflective coaching behaviours, and underscored the importance of the use of positive language, active and engaged listening and relational strategies (King et al., 2023). Furthermore, these same authors also identified key parent/client change mechanisms resulting from these approaches which were linked to parent/client engagement and self-efficacy (King et al., 2023).

Telehealth has been reported to promote therapist-coaching behaviors due to the therapist's lack of physical proximity to the child (Kwok et al., 2022; Olsen et al., 2012; Retamal-Walter et al., 2022). Positive telehealth experiences for parents have been reported when therapists use coaching-style communication (Graham et al., 2023). Yet therapists' narratives suggested that building the necessary engagement and rapport with parents and children is more challenging in telehealth (Akamoglu et al., 2018; Fishman & Elkins, 2022; Hall et al., 2021; Hines et al., 2019; Retamal-Walter et al., 2022; Yoo et al., 2021). In the recent evaluation of a newly developed fidelity instrument (i.e., CO-FIDEL) used to assess a different telehealth coaching intervention (i.e., Bright-Coaching), study participants highlighted that engagement and rapport were missing elements from the measure and recommended their addition in future versions (Ogourtsova, Majnemer, et al., 2023). Owing to the therapist's reliance on the parent to carry out the tasks, studies have suggested that higher levels of parent engagement are required in telehealth interventions than in those delivered in-person, and that such engagement is vital to the success of pediatric telehealth interventions (Hall et al., 2021; Kwok et al., 2022; Pozniak et al., 2023; Retamal-Walter et al., 2022; Yoo et al., 2021).

An international expert panel identified 64 behaviors deemed critical for building engagement in pediatric telehealth (Retamal-Walter et al., 2022). Like in the *WECARE Fidelity Checklist*, these experts identified a higher number of therapists' behaviors than those attributed to parents (Retamal-Walter et al., 2022). Furthermore, like previously published literature (Akamoglu et al., 2018; Hines et al., 2019) and as reflected in our checklist, Retamal-Walters and colleagues (2022) also underscored the importance of collaborative behaviors, effective communication, and behaviors acknowledging families (e.g., actively listening to family; discussing therapy objectives; co-designing intervention strategies). In addition to building engagement, evidence suggests that these therapist's behaviors can also boost parent's confidence and competence, improve their well-being and their self-efficacy in their parenting role, and empower them to direct their child's intervention, regardless of the mode of service delivery (i.e., in-person or telehealth) (King et al., 2023; Tkach & Earwood, 2023).

While similarities between telehealth and in-person engagement strategies exist, the rapid proliferation and adoption of telehealth in clinical practice, and the subsequent research have recently led to the identification of telehealth-specific engagement behaviors, which had yet to be recognized when the *WECARE Fidelity Checklist* was developed. For example, studies by Retamal-Walters and colleagues (2022; 2023) and Kwok and colleagues (2022) identified therapist, family, and therapist-parent interactive behaviors specific to successful engagement in telehealth. Therapists' behaviors included: adjustments to professionals' communication style to encompass comments on the family's environment, using resources from the family's home during the session, maximizing visual and auditory channels (e.g., using visual aids, providing detailed verbal and/or written instructions), being animated throughout the telehealth session (Retamal-Walter et al., 2022), as well as therapists' calmness, flexibility and openness (Kwok et al., 2022; Retamal-Walter et al., 2023). Child, parent and additional family member behaviors and therapist-parent interactive behaviors encompass deliberately getting to know each other, being responsive, open and honest with therapists, using a natural communication style, parent's understanding and capacity to support the child during the intervention, joint planning, relationship development outside telehealth sessions, and embracing the home environment (Kwok et al., 2022; Retamal-Walter et al., 2023). Although some of these behaviors may not seem unique to telehealth, therapists may need to address them with more intentionality in the telehealth context than during in-person interactions, particularly early in the intervention process, when building trust and buy-in with families are crucial (Retamal-Walter et al., 2023).

As such, in our study, raters recommended a review of the checklist domains, suggesting a categorization or matching of the behaviors to specific intervention phases (e.g., initial assessments vs. follow-up or monitoring visits). Retamal-Walter and his colleagues (2023) found that, for example, a therapist's experience with technology may not be vital for family engagement during the telehealth intervention yet they hypothesized it to be critical when first deciding on the mode of service delivery (e.g., telehealth, in-person, hybrid). Although these newly identified telehealth-specific engagement behaviors and their categorization to specific phases of the intervention may be helpful, before making any of these suggested changes to our checklist, careful examination is needed to explore how these newly identified behaviors apply to the *WECARE* intervention. Furthermore, additional research may be required to investigate parent (and child) engagement across the intervention continuum, and if the specific behaviors which foster engagement in initial telehealth encounters differ from those required in follow-up intervention or monitoring sessions in this mode of delivery.

### Phase 2. Implementation Assessment

In addition to detailing the development process, we also sought to assess the implementation of the *WECARE Fidelity Checklist* by estimating its inter-rater reliability and describing the raters' initial experience with the tool as a means of identifying checklist improvement and refinement.

Although raters' satisfaction, confidence in scores, and their perceived ease of use of the measure scored high, they did report variability in the video quality (e.g., low audio, background noise, absence of video, unable to see parent and therapists on screen at the same time), queried the focus of some videoed sessions which were more assessment- rather than intervention-based, and highlighted scoring difficulty due to the uncertainty and lack of clarity of the session objective being addressed. Similar procedural issues were raised in a study examining fidelity in pediatric rehabilitation (DiRezze et al., 2013), and those using video-recordings (Ogourtsova, Majnemer, et al., 2023; DiRezze et al., 2013). The comments from the raters in our study suggest that these procedural issues can complicate the use of the measure and its measurement system. Moreover, raters in our study suggested that a standardized procedure be implemented to ensure high-quality recordings are available to raters for scoring.

The inclusion of additional ratings was also suggested by the raters in our study. For example, a “not applicable” (e.g., behavior observed is not appropriate for the type of intervention), and/or “unable to score” (e.g., audio only when video required, parent and therapists are not visible on the screen at the same time) were recommended as possible rating additions to the measurement coding system.

In the estimation of the inter-rater reliability of the *WECARE Fidelity Checklist,* the tool demonstrated moderate reliability scores in two subscales (i.e., therapist and parent-therapist interactions); however, the large confidence intervals, indicated considerable uncertainty which cannot be ignored. The reliability estimates are consistent with initial levels of inter-rater agreement regarding fidelity in other complex interventions (Ginsburg et al., 2021; Walton et al., 2020; Walton et al., 2019).

The variations found in our study could be explained by many factors including a lack of standardized training in using the measure, limited understanding of the behaviors being assessed, and the complex measurement coding system. While the *WECARE Fidelity Checklist* was developed and definitions were reviewed and refined during the development process with Rater 1 and 2, Rater 3 was not provided with the same opportunity. Furthermore, the informal training process provided to Rater 3 (approximately 2 hours in duration), her limited knowledge of coaching strategies and her limited clinical experience may have impacted her understanding of the target behaviors of the *WECARE Fidelity Checklist* measurement system. Upwards of 16 hours of training, comprised of didactic instruction, rating of video-recorded intervention and the gathering of regular feedback about encountered difficulties have been recommended in previous studies, especially when complex scoring measurement systems are used (An et al., 2021; Carroll et al., 2007; DiRezze et al., 2013). Furthermore, previous literature has advocated for a minimum amount of clinical experience (i.e., > 5 years) for raters due to the complexity of the rating tasks, including the behaviors being evaluated and the complexity of the measurement system (Elvins & Green, 2008; DiRezze et al., 2013).

A complex measurement system can create challenges, especially when raters are familiarizing themselves with the system, or for those with limited experience with coaching approaches. Our raters and those in other studies (DiRezze et al., 2012) underscored that raters' comfort level with the measurement system requires time and practice; as more videos were scored, awareness increased as to what to look for in the recorded session. Evidence suggests that rating practice should include at least 10 videos and feedback should be provided by an expert (e.g., measurement developer or expert panel) on the scoring (Carroll et al., 2000; Martino et al., 2008; DiRezze et al., 2013). An acceptable agreement level (e.g., 75%) in terms of correct rating should be chosen a priori, and evaluated until achieved by each rater, with remediation provided for a rater should the agreement level not be achieved (DiRezze et al., 2013). Furthermore, to ensure the maintenance of a standard level of scoring post-training, the implementation of a quality assurance process (e.g., an expert reviewing the rating of random sessions at various time points) is also strongly suggested to monitor rater drift, with remediation provided should such drift be observed (Martino et al., 2008). Raters' experience survey comments underscored concerns associated with rater drift, specifically, when returning to rating after a hiatus.

The 5-point Likert measurement system chosen for scoring the subdomains items in the *WECARE Fidelity Checklist* not only considered the presence or absence of behaviors, but it also assessed whether they occurred at the appropriate frequency and time during the session. In the development of a generic fidelity measure for pediatric rehabilitation, DiRezze and colleagues (2013) noted that their scoring system assumed that a high frequency of a behavior translated to higher fidelity, and the “appropriateness” of the behavior was not considered. A recently developed fidelity measure for a coaching intervention (Graham et al., 2020) underscored the importance of appropriate timing of behaviors, and that all potential opportunities to implement them are used. As a result, the “appropriateness” of the observed behavior was included in our scoring definitions. However, raters did underscore the complexity of the rating system. Future iterations of the *WECARE Fidelity Checklist* may also consider separating the dimension of frequency (i.e., observation of the behavior) and quality (i.e., behavior observed when appropriate, and not observed when not appropriate), assessing what correlation (if any) exists between the dimensions, and whether only quality (DiRezze et al., 2013) or both dimensions should be evaluated in determining the fidelity of the *WECARE* intervention.

Finally, raters' comments highlighted two items that require rewording. Large variations were noted in reliability on item 4 (i.e., *The therapist agrees with the parent-identified centered objectives that will guide the session*) and item 10 (i.e., *The therapist gives advice, information or references, only when asked by the parent to do so*). More specifically the raters highlighted that the item descriptions contradicted the evidence related to family-professional collaboration, a cornerstone of family-centered care practices and pediatric rehabilitation (An & Palisano, 2014). Family–professional collaboration is a mutually supportive interactive process where families and providers share knowledge and skills, joint understanding occurs, and shared decisions result associated with identified goals, and intervention planning, which address the family's concerns, needs and priorities (An & Palisano, 2014; An et al., 2016; Rosenbaum et al., 2014). The current wording of the items fails to acknowledge the value parents attribute to provider input in this collaborative process of goal-setting and planning (Pritchard-Wiart et al., 2019). Studies examining the importance parents attribute to therapists' input emphasize that many families prefer not to have the exclusive responsibility of identifying treatment objectives and underscore the benefits of a co-creation process for goal development and treatment planning grounded in the family's concerns within their context (Forsingdal et al., 2014; Øien et al., 2010; Wiart et al., 2010).

### Study Limitations and Future Research

Several limitations were evident in this study. In Phase 1, the behaviors included in the *WECARE Fidelity Checklist* were based on the *WECARE* intervention theory of change, which had yet to be evaluated. Moreover, the *WECARE* intervention was composed of multiple telehealth components, which included but were not limited to parent-therapist coaching sessions via telehealth. This study only piloted the application of the checklist to the videoconferencing component of the intervention; the other components (e.g., private messaging feature) were not evaluated. In addition to the theory of change, the behavior items identification process also relied on a rapid scan of the literature and expert (n=2) opinion. A more in-depth review of the literature (e.g., systematic review (Ogourtsova, Boychuck, et al., 2023; Ogourtsova, O'Donnell, et al., 2019)) and/or a broader consultative process (e.g., Delphi technique (Retamal-Walter et al., 2022)) may have identified additional behaviors for which inclusion would enhance the domains (e.g., parent-therapists interaction). Finally, no item explored the impact of technology issues on the fidelity of a telehealth intervention delivery. Recent studies, as well as our raters' comments, highlight issues with technology as important deterrents to telehealth service delivery (De Taeye et al., 2023; Dostie et al., 2023; Grant et al., 2022; Hall et al., 2022; Hall et al., 2021; Kwok et al., 2022; Rettinger & Kuhn, 2023; Tully et al., 2021; Wittmeier et al., 2022). Consequently, the inclusion of behaviors related to navigating technology and/or associated with partnering with parents to resolve technological issues may need to be considered when assessing the fidelity of telehealth interventions.

The assessment of the implementation of the *WECARE Fidelity Checklist* tool (i.e., Phase 2) provided many insights for its refinement and future use. While the reasons for our implementation assessment results are unclear, addressing rater training and the ambiguity of scoring criteria by applying the insights reported by our external raters are recommended for future research. More specifically, the additional ratings (i.e., not applicable; unable to score) suggested by raters should be included and the problematic items (i.e., items 4 and 10) should be reworded to align with current evidence. A user manual should be developed with key definitions and observable behaviors descriptions for each item with examples. The rationale for scoring related to both rating dimensions (e.g., frequency and appropriateness) should be provided and illustrated through scenarios. The revised instrument and manual should then be examined and refined through a series of meetings with either those involved in the instrument development and the external raters participating in this initial testing phase or an expert panel. Consensus methodology (e.g., Delphi technique, Nominal Group technique, TRIAGE) could be used to systematically guide the process.

During these discussions, content for upwards of 16 hours of formal training could also be identified. Rating observations and discussion, as well as scoring practice on at least 10 high-quality videos which would be assessed against expert consensus should be included. This ‘expert’ group should also determine the level of acceptable agreement in terms of the proportion of codes rated correctly for each domain and how ‘agreement’ is defined. The training and rating practice should be condensed into a predetermined period (e.g., 3 weeks) in which raters independently score video recordings. Evaluation of raters should continue until each rater reaches the acceptable a priori set agreement level. Remediation in the form of a videoconferencing meeting to discuss scoring rationale, guided by the developed user manual could be provided if the agreement level is not reached.

Once the instrument is refined and the training process is determined, at least six raters should pilot the training and data should be gathered to evaluate it. Consideration should be given to the criteria used for rater selection. Not only should attention be given to years of pediatric clinical experience, but their knowledge of coaching behaviors and telehealth practice should be considered. Given the complex behaviors associated with this approach, further training (e.g., scoring practice) and support (e.g., external feedback) may be required for those less familiar with the approach to ensure that scoring discrepancies are identified early and addressed promptly.

When the training is completed and the predetermined level of agreement is achieved, raters could be asked to score a set sample of high-quality video recordings of sessions. Ongoing rater monitoring and support, whether self-assessment or via external feedback, should also be pondered to address deviations (i.e., rater drift) and to ensure correction occurs in a timely fashion if they do occur. This process would not only allow for the re-testing of interrater reliability but also the evaluation of other psychometric properties (e.g., intra-rater reliability).

Should a sufficient level of reliability be obtained, and a large enough sample of quality video recordings be available, applying item response theory to the data to further evaluate the items in the instrument could be explored. A larger sample of raters would allow for the examination of raters' disciplines (e.g., occupational therapists and physiotherapists) and other rater characteristics (e.g., year of clinical experience; coaching and telehealth experience) on scoring, and to further explore whether the multitude of factors (i.e., frequency and “appropriateness” of behaviors) are required to assess quality ratings in each subdomain. Finally, although the *WECARE fidelity checklist* was designed as an intervention-specific instrument, given the dearth of available measures to assess the delivery of coaching interventions using telehealth, examining its application to coaching approaches, other than *WECARE*, in research and practice may also be worth exploring in future studies.

## Conclusion

Findings from this study demonstrate the value of using a structured approach in developing an intervention-specific fidelity measure to ensure that the key ingredients of a complex intervention are accurately assessed. Furthermore, it highlights the benefit of assessing the implementation of new tools by collecting raters' experiences in this early phase. Not only did these data provide clarity as to why certain measurement items may be problematic, but they also identified areas of refinement in the measurement coding system, enhancements to the associated rating procedure, including initial and ongoing standardized training for raters using the measure, and criteria upon which to judge the quality of the video recordings employed in future formalized reliability studies. Once refined and further studied, the *WECARE Fidelity Checklist* may be useful in clinician and student training, in the evaluation of family-centered pediatric telehealth coaching interventions. The tool may also be of assistance in determining whether therapists have received adequate training and support to deliver these interventions. Finally, the process of development and piloting undertaken in this study may be valuable to others planning to evaluate the fidelity of complex telehealth interventions.
